# Frequency and risk factors of sleep problems in Egyptian patients with multiple sclerosis

**DOI:** 10.3389/fneur.2025.1563041

**Published:** 2025-04-30

**Authors:** Eman M. Khedr, Gellan K. Ahmed, Shady Safwat Hassan, Mohamed Nageh Foly, Motez Mahmoud Attia, Ahmed A. Karim, Nourelhoda A. Haridy

**Affiliations:** ^1^Neurology and Psychiatry Department, Assiut University, Assiut, Egypt; ^2^Neurology and Psychiatry Department, Aswan University, Aswan, Egypt; ^3^Assiut University Hospitals, Assiut, Egypt; ^4^Department of Psychiatry and Psychotherapy, University of Tübingen, Tübingen, Germany

**Keywords:** multiple sclerosis, sleep quality, quality of life, fatigue, Pittsburgh Sleep Quality Index

## Abstract

**Background:**

Sleep problems impact over 65% of patients with multiple sclerosis (MS), a prevalence significantly greater than that observed in the general population. This study aimed to assess the frequency and risk-associated factors of sleep problems in a large MS cohort and evaluate their impact on quality of life (QoL).

**Methods:**

The study included 103 participants with MS across different disease stages and 62 healthy controls. Assessment tools included the Expanded Disability Status Scale (EDSS), Pittsburgh Sleep Quality Index (PSQI), depression and fatigue scales, 9-Hole Peg Test, 25-foot walk test, cognitive function assessments, and QoL measures.

**Results:**

Sleep problems were significantly more frequent in MS patients (68.9%) than in controls (30.6%). PSQI scores showed positive correlations with the number of MS relapses across the course of disease duration, walking impairment, fatigue and depression scores. Sleep problems were determined to adversely affect various domains of quality of life.

**Conclusion:**

Our findings demonstrate that sleep problems are remarkably common among patients with MS. Patients experiencing poor sleep quality are typically associated with higher levels of fatigue, depression, greater difficulty with mobility, and more frequent disease relapses. These sleep problems significantly impaired the overall QoL in MS patients. A multidisciplinary approach is therefore essential for managing sleep disorders in MS.

## 1 Introduction

Multiple sclerosis (MS) is an autoimmune illness that mainly affects young individuals (1). The prevalence ranges from 50 to 300/100,000 persons (1). The disease’s characteristics include neurological symptoms that are exacerbated during attack periods, including weakness, vertigo, numbness, blindness, cognitive changes, and sleep difficulties (2). It leads to a wide range of neurological symptoms, including fatigue, cognitive impairment, and sleep disturbances. Studies have reported that 25%–67% of MS patients experience poor sleep quality, a rate significantly higher than in the general population (3). Sleep problems in MS include insomnia, restless legs syndrome (RLS), narcolepsy, and obstructive sleep apnea (OSA), all of which contribute to worsening fatigue, depression, and overall disease burden (4).

Sleep and MS have a complex and reciprocal interaction. The disease process in MS may disrupt neural breathing centers, causing ventilatory abnormalities during both waking and sleeping states (5). Furthermore, various MS-associated symptoms, including muscular spasms, nocturia, and fatigue, were identified as primary contributors to sleep disturbance in MS (6). Additionally, sleep disturbances may be precipitated by the severity of the disease, comorbidities, and associated adverse effects of therapy (4).

Sleep disorders, in turn, can intensify MS symptomatology, resulting in numerous debilitating conditions during wakefulness, including cephalgia, chronic pain, fatigue, depression, and cognitive dysfunction (4). Additionally, sleep disturbance has been implicated as a potential trigger for acute MS exacerbations (7). Recent research suggests that compromised sleep problems and insufficient duration of sleep during adolescence may constitute risk factors for MS development (8). This intricate interaction significantly diminishes the quality of life (QoL) of individuals with MS, with manifestations such as daytime somnolence and insomnia demonstrating substantial adverse effects on patient well-being (9).

Recent research underscores the imperative for thorough sleep analysis in MS patients. While polysomnography (PSG) is the definitive diagnostic method for sleep disorders, detailed sleep questionnaires can potentially aid early MS detection. For diagnosed MS patients, independent sleep disorder evaluations are essential, as addressing sleep issues may significantly improve overall MS symptoms (10).

While previous studies have examined sleep disturbances in MS, most have focused on Western (6) or East Asian populations (3), leaving a gap in understanding how geographic, cultural, and demographic factors influence sleep quality in MS. The present study is among the first to comprehensively evaluate sleep problems in an Egyptian MS cohort, providing novel insights into how regional differences in sleep habits, climate, and healthcare access may contribute to variations in sleep quality among MS patients.

In addition to its geographic focus, this study is methodologically distinct from many prior investigations. Several factors set our study apart: Unlike retrospective registry-based studies, our cross-sectional design ensured structured, supervised data collection at a specialized MS unit, reducing selection and recall biases. While many studies primarily examine relapsing–remitting MS (RRMS), our study includes a broad range of MS phenotypes (CIS, RRMS, SPMS, and PPMS), allowing for a more comprehensive analysis of sleep disturbances across different disease stages. Comprehensive sleep and clinical assessments: Previous research often relied on a single sleep assessment tool. In contrast, our study utilizes the Pittsburgh Sleep Quality Index (PSQI) and correlates sleep findings with: Neurological disability (EDSS, 25-FWT, 9-HPT), Fatigue (MFIS), Depression (HDRS), Cognitive function (SDMT, BVMT-R, CVLT-II) and Quality of life (SF-36). Focus on Disease-Modifying Therapies (DMTs): While some studies overlook the potential influence of MS treatments on sleep, our study stratifies patients based on DMT use and efficacy levels, offering valuable insights into possible medication effects on sleep quality.

Given these methodological advancements and the unique characteristics of our study population, we aimed to assess the frequency of sleep disturbances in Egyptian MS patients, explore their associations with clinical and cognitive variables, and investigate the impact of sleep quality on quality of life.

## 2 Methods

### 2.1 Study design

This cross-sectional study was carried out at Al-Eman MS Unit, Nile Valley, Assiut governorate, Upper Egypt. All confirmed MS patients who attended the unit for either diagnosis or during follow-up visits from January 1, 2023, to October 31, 2024, were consecutively recruited. At our center, MS patients typically undergo follow-up visits every 3 months, allowing for systematic recruitment over time.

The study was conducted in compliance with the Declaration of Helsinki and approved by the Institutional Review Board’s ethical committee at the Assiut University Faculty of Medicine (IRB: 04-2025-300553). Before taking part in the trial, each subject gave written informed consent.

### 2.2 Sample size calculation

Sample size calculation used G*Power 3.1 (11, 12), based on Mosarrezaii et al. (13) finding that 69.1% of MS patients had poor sleep quality. With medium effect size (d = 0.5), *α* = 0.05, and power = 0.80, we needed 64 participants per group (128 total), and 100 MS patients. We were able to recruit 165 participants (103 MS patients, and 62 healthy controls).

### 2.3 Participants

According to the 2017 revised McDonald criteria (14), a total of 200 MS patients were invited to participate, of whom 103 patients were enrolled, encompassing various stages of the disease. The remaining 97 patients were not included due to either refusal to participate (30 patients) as they came from areas away from the hospital or incomplete data (67 patients). Furthermore, 62 healthy matched controls, aligned for age and sex distribution, were recruited. These controls were unconnected to MS patients and free from medical illnesses or drugs. All participants completed the study questionnaires at the MS Unit under supervision, ensuring consistency in data collection.

*Inclusion criteria:* Between 18 and 55 years old, both sexes were included, and no evidence of relapse either clinically or radiologically during the 3 months before participating in the study. *Exclusion Criteria:* Patients who had systemic disease, psychiatric disorders, and body mass index (BMI) ≥ 30 were excluded due to the higher prevalence of sleep apnea, which could confound fatigue assessment, or on long-term treatment other than DMT, such as antipsychotics and antidepressants or substance abuse. Pregnant and lactating patients were also excluded.

Various MS phenotypes categorized patients into four groups: Clinically Isolated syndrome (CIS), Relapsing–Remitting (RRMS), Secondary (SPMS), and Primary Progressive Multiple Sclerosis (PPMS) (1, 14). We included CIS patients according to the McDonald criteria 2017 (14), as they added the following changes: in patients with a typical CIS and clinical or MRI demonstration of dissemination in space, the presence of CSF-specific oligoclonal bands allows a diagnosis of MS and all participating patients had positive OCB and had definite MS diagnosis. PPMS and SPMS were classified as Progressive MS, while CIS and RRMS were combined into a non-progressive MS group. Depending on their DMT status, patients were classified as DMT-Naïve, having taken DMT for less than 12 months or receiving DMT for more than 12 months.

The 12 months was selected because it is widely acknowledged as the time frame after which most DMTs become effective (15). The patients in the treated group were allocated to one of three DMT groups based on their level of efficacy: low (Interferons, Dimethyl fumarate, and Teriflunomide), moderate (Fingolimod), and high (Ocrevus and Rituximab).

### 2.4 Clinical assessment

Participants underwent a comprehensive evaluation, including demographic data collection and clinical characteristics of MS patients. MS phenotype (CIS, PPMS, RRMS, SPMS), and treatment status (drug-naïve vs. on disease-modifying therapies). The current Expanded Disability Status Scale (EDSS), 9-Hole Peg Test (9-HPT), 25-foot-walk test (25-FWT), Hamilton Depression Rating Scale (HDRS), and Pittsburgh Sleep Quality Index (PSQI), were assessed. Cognition assessment scales included the California Verbal Learning Test-II (CVLT-II), Brief Visuospatial Memory Test (BVMT), and Symbol Digit Modalities Test (SDMT). Revision of all available radiological examinations for the presence of any new radiological activity and lesion distribution were recorded. Laboratory tests were performed, including the number of oligoclonal bands in the cerebrospinal fluid (CSF OCB), and sNfL levels in the serum were reported at the same examination visit.

#### 2.4.1 Expanded Disability Status Scale

This test measures MS-related disability. It assesses eight functional systems, with scores in 0.5-point increments. Lower scores indicate mild neurological disability, while higher scores (>6) represent more severe disabilities. Walking ability is crucial, especially between EDSS 4–6 (16). EDSS is the internationally accepted standard for MS outcome measurement in clinical trials, enabling cross-study comparisons despite limitations (17).

#### 2.4.2 Pittsburgh Sleep Quality Index

This self-reported questionnaire is used to evaluate sleep quality over 1 month. The seven dimensions of sleep include subjective sleep quality, latency, length, habitual efficiency, interruptions, sleeping medication use, and dysfunction during the day. Scores are assigned on a scale from 0 to 21, where higher values signify a reduction in the quality of sleep (18). A score of five or higher indicates substantial difficulty in more than three subscales or significant sleep disturbances in at least two subscales. The PSQI has internal consistency and a reliability coefficient (Cronbach’s alpha) of 0.83 for its seven components (18).

#### 2.4.3 The Modified Fatigue Impact Scale

The Modified Fatigue Impact Scale (MFIS) was validated by Kos et al. (19). This 21-item test evaluates three aspects of fatigue: psychological, physical, and cognitive. The overall score is standardized on a 100-point scale from 0 to 84. Pathological weariness is indicated by a score of ≥45/100 (20). MFIS has high reliability with Cronbach’s alpha equal 0.90 (21).

#### 2.4.4 Hamilton Depression Rating Scale

A 17-item measure of the intensity of depression. It evaluates symptoms like anxiety, agitation, weight loss, sleeplessness, and bad mood. There are several levels of depression: mild (8–13), moderate (14–18), severe (19–22), extremely severe (23–≥23), and normal (0–7) (22). A meta-analysis (409 studies) shows the reliability of the HDRS: internal consistency 0.784 (CI: 0.778–0.789), inter-rater ICC 0.942 (CI: 0.938–0.947), test–retest ICC 0.93 (CI: 0.88–0.96) (23).

#### 2.4.5 Short-Form Health survey

The Short-Form Health survey (SF-36), a comprehensive psychometric instrument, was developed by the Rand Corporation in the 1970s. It consists of a 36-item questionnaire that assesses health status and quality of life across eight domains: physical functioning, limitation of physical health, limitation of emotion, social functioning, body pain, emotion wellbeing, energy/fatigue, and general health perceptions. Scores are assigned to each domain on a scale of 0–100, with higher scores indicating improved health. Physical (PCS) and mental (MCS) component scores can be used to summarize the results (24). The overall Cronbach’s alpha typically ranges ranged from 0.72 to 0.95 (25). A validated Arabic version was used (26).

#### 2.4.6 9-Hole Peg Test

The test protocol requires sequential placement and removal of nine pegs into corresponding holes, with various apparatus designs available (27, 28). While completion time in seconds remains the predominant metric, recent studies have adopted pegs per second (pegs/s) as an alternative measure. This metric, calculated from completed pegs within the standard time or placed within a 300-s limit, addresses floor effects in severe upper limb dysfunction and facilitates normal data distribution for statistical analyses. Standard administration involves four trials, with two trials per hand (29). The 9-HPT has excellent test–retest reliability intraclass correlation coefficients (ICC) = 0.947 for the non-dominant hand, ICC = 0.937 for the dominant hand (30).

#### 2.4.7 25-foot-walk test

It is the most commonly used validated assessment tool for quantifying ambulatory function (31). While frequently administered as an MS Functional Composite (MSFC) component, it has demonstrated utility as an independent measure in clinical investigations. The standardized administration protocol requires patients to traverse a precisely demarcated 25-foot course bidirectionally at a maximum safe velocity. Temporal measurement commences upon instructional initiation and terminates upon crossing the designated endpoint. Two consecutive trials are conducted immediately, with the return traverse as the second trial. The protocol permits utilizing ambulatory assistive devices during test execution (32). The T25FW measure demonstrated excellent test–retest reliability (ICC = 0.98) (33).

#### 2.4.8 Cognitive assessment tools

The cognitive assessment battery administered to all MS patients comprised three standardized instruments: The CVLT-II, which evaluates verbal learning and memory functions (34); the SDMT, which evaluates the capacity of working memory and the efficiency of information processing (35); and the BVMT-R, used to assess visuospatial learning and memory functions (36). Cognitive impairment classification utilized established threshold values as defined by Khedr et al. (37): BVMT-R ≤ 10, SDMT ≤22, and CVLT-II ≤ 38. Following Benedict et al.’s (38) criteria, cognitive impairment was determined by performing below the threshold on at least two of the three administered tests. The intra-observer (test–retest) reliability was satisfactory for SDMT, CVLT-II, and BVRT-R with *r* values of 0.85, 0.61, and 0.68, respectively based on the Arabic validation of these tests. Validated Arabic versions were used (39).

### 2.5 Statistical analysis

For the analysis of the data, SPSS version 26 was utilized. The Shapiro–Wilk test, which confirmed the non-normal data distribution, served as a reference for the use of non-parametric statistical methods. For continuous data, the median, interquartile range (IQR), and mean ± standard deviation (SD) were shown. Categorical data were represented using percentages and frequencies. The mean values of the continuous variables were compared using the Mann–Whitney U tests. The category variables were compared using the chi-square or Fisher’s exact test. The Spearman’s rank correlation coefficient was used to evaluate the association between PSQI scores and other clinical and demographic factors. Statistical significance was defined as a *p*-value of less than 0.05.

## 3 Results

The demographic details and sleep quality of MS patients and controls are shown in Table 1. Compared to healthy controls (*n* = 62), MS patients (*n* = 103) experienced considerably worse sleep quality (68.9% vs. 30.6%, *p* = 0.0001). Additionally, the MS group’s mean PSQI score was substantially higher (7.97 ± 4.8) than that of the control group (4.09 ± 3.39, *p* = 0.001), suggesting that MS patients have poorer sleep quality.

**Table 1:** Demographic and sleep disorders (using Pittsburgh Sleep Quality Index) among studied groups.

	MS group (*n* = 103)	Healthy control group (*n* = 62)	*p* value
Age			
Mean ± SD	31.54 ± 8.75	31.13 ± 13.02	0.214
Median (IQR)	31 (15)	26.5 (26)	
Sex			
Males	28 (27.2%)	19 (30.6%)	0.722
Females	75 (72.8%)	43 (69.4%)	
Total PSQI score			
Mean ± SD	7.97 ± 4.8	4.09 ± 3.39	0.001
Median (IQR)	8 (7)	3 (4)	
Good sleep quality	32 (31.1%)	43 (69.4%)	0.0001
Poor sleep quality	71 (68.9%)	19 (30.6%)	
Time to sleep (min)	35.70 ± 36.43	22.32 ± 22.71	0.313
Sleep hours	6.63 ± 2.06	6.97 ± 1.83	0.338
PSQI subscales			
Subjective sleep quality	1.17 ± 0.81	0.97 ± 0.80	0.179
Sleep latency	1.42 ± 1.00	1.03 ± 0.84	0.046
Sleep duration	1.29 ± 1.17	1.19 ± 1.14	0.675
Habitual sleep	1.31 ± 1.21	0.45 ± 0.93	<0.001
Sleep disturbances	1.38 ± 0.69	1.06 ± 0.63	0.039
Use of sleep medication	0.42 ± 0.98	0.26 ± 0.77	0.452
Daytime dysfunction	0.92 ± 1.75	0.94 ± 0.96	0.282

The Chi-square test was used for categorical variables. The Mann–Whitney test was used for continuous variables. IQR, interquartile range; MS, multiple sclerosis; PSQI, Pittsburgh sleep quality index; SD, standard deviation.

Table 2 compares the demographic characteristics of MS patients with poor sleep quality (*n* = 71) to those with good sleep quality (*n* = 32). There were no statistically significant differences between the two groups in age, sex distribution, years of education, occupation, marital status, smoking, or any medical comorbidities.

**Table 2:** Demographic data in relation to quality of sleep (Poor vs. good quality sleep) according to PSQI among MS patients.

	Poor sleep quality (*n* = 71 cases)	Good sleep quality (*n* = 32cases)	Total MS population (*n* = 103)	*p*-value
Age				
Mean ± SD	31.83 ± 9.06	30.84 ± 7.95	31.52 ± 8.7	0.67
Median (IQR)	30 (16)	31 (15)	31 (15)	
Sex				
Males	17 (23.9%)	11 (34.4%)	28 (27.2%)	0.33
Females	54 (76.1%)	21 (65.6%)	75 (72.8%)	
Education years				
Mean ± SD	10.4 ± 4.59	11.09 ± 4.18	10.64 ± 4.46	
Median (IQR)	12 (3)	12 (3)	12 (5)	0.52
Occupation				
Working	15 (21.1%)	12 (37.5%)	27 (26.2%)	0.094
Non-working	56 (78.9%)	20 (62.5%)	76 (73.8%)	
Marital status				
Married	46 (64.8%)	20 (62.5%)	66 (64.1%)	0.82
Single	25 (35.2%)	12 (37.5%)	37 (35.9%)	
Smoking	4 (5.6%)	3 (9.4%)	7 (6.8%)	0.67
Other medical comorbidity	5 (7%)	4 (12.5%)	9 (8.7%)	0.45
Time to sleep (min)	12.90 ± 11.72	46.09 ± 39.12	35.70 ± 36.43	<0.001
Sleep hours	8.10 ± 1.44	5.96 ± 1.96	6.63 ± 2.06	<0.001
PSQI subscales	2.88 ± 1.19	10.27 ± 3.98	7.97 ± 4.81	<0.001
Subjective sleep quality	0.59 ± 0.50	1.44 ± 0.79	1.17 ± 0.81	<0.001
Sleep latency	0.47 ± 0.67	1.85 ± 0.80	1.42 ± 1.00	<0.001
Sleep duration	0.50 ± 0.88	1.65 ± 1.11	1.29 ± 1.17	<0.001
Habitual sleep	0.22 ± 0.49	1.80 ± 1.10	1.31 ± 1.21	<0.001
Sleep disturbances	1.00 ± 0.44	1.55 ± 0.71	1.38 ± 0.69	<0.001
Use of sleep medication	0.00 ± 0.00	0.61 ± 1.13	0.42 ± 0.98	0.001
Daytime dysfunction	0.13 ± 0.49	1.28 ± 1.98	0.92 ± 1.75	<0.001
PSQI total score	2.88 ± 1.19	10.27 ± 3.98	7.97 ± 4.81	<0.001

The Chi-square test was used for categorical variables. The Mann–Whitney test was used for continuous variables. IQR, interquartile range; PSQI, Pittsburgh sleep quality index; SD, standard deviation.

Table 3 shows sleep quality and various clinical characteristics in MS patients. RRMS was the most common phenotype in both groups (67.6% in poor sleep quality, 75% in good sleep quality). Interestingly, all patients with SPMS fell into the poor sleep quality group, although the overall distribution of phenotypes showed no differences between groups (*p* = 0.219). The majority of patients in both groups were drug-naïve (80.3% in poor sleep quality, 78.1% in good sleep quality). Notably, patients with poor sleep quality (*n* = 71) demonstrated significantly higher total number of attacks across the whole disease duration (*p* = 0.04), worse performance on the 25-FWT (*p* = 0.029), greater fatigue on the MFIS (*p* = 0.03), and higher depression scores on the HDRS (*p* = 0.027) compared to those with good sleep quality (*n* = 32). Demographics, clinical presentation, MRI findings, cognitive function, and others show no significant differences between poor and good sleep quality were observed.

**Table 3:** Associations between poor sleep according to DMS5 and non-parametric clinical data.

	Poor sleep quality (*n* = 71)	Good sleep quality (*n* = 32)	Total MS population (*n* = 103)	*p*-value
Clinical phenotype				
Clinically isolated syndrome (CIS)	11 (15.5%)	7 (21.9%)	18 (17.5%)	0.219
Primary progressive multiple sclerosis (PPMS)	5 (7%)	1 (3.1%)	6 (5.8%)	
Relapsing–remitting multiple sclerosis (RRMS)	48 (67.6%)	24 (75%)	72 (69.9%)	
Secondary progressive multiple sclerosis (SPMS)	7 (9.9%)	0 (0%)	7 (6.8%)	
Treated vs. non-treated patients				
Drug Naïve MS patients	57 (80.3%)	25 (78.1%)	82 (79.6%)	0.79
MS patients under DMTs	14 (19.7%)	7 (21.9%)	21 (20.4%)	
Clinical criteria				
Disease duration (years)	4.51 ± 4.23	3.85 ± 3.4	4.30 ± 3.98	0.78
Total number of attacks	3.27 ± 2.11	2.53 ± 1.75	3.04 ± 2.02	
Median (IQR)	3 (2)	2 (2)	3 (3)	0.04
First clinical presentation				
Motor	26 (36.2%)	9 (28.1%)	35 (34%)	0.5
Sensory	14 (19.7%)	11 (34.4%)	25 (24.3%)	0.13
Optic	17 (23.9%)	10 (31.3%)	27 (26.2%)	0.47
Cerebellum	8 (11.3%)	2 (6.3%)	10 (9.7%)	0.72
Other	6 (8.5%)	0 (0%)	6 (5.8%)	0.17
MRI findings				
Periventricular	61 (85.9%)	26 (81.3%)	87 (84.5%)	0.56
Juxta cortical	49 (69%)	20 (62.5%)	69 (67%)	0.65
Infra tentorial	18 (25.4%)	6 (18.8%)	24 (23.3%)	0.61
Spine	23 (32.4%)	15 (46.9%)	38 (36.9%)	0.18
Severity				
EDSS	1.97 ± 1.17	1.57 ± 0.87	1.85 ± 1.1	0.119
Median (IQR)	1.5 (1.5)	1.5 (1)	1.5 (1)	
Motor disability and fatigue assessment				
25-FWT	18 ± 6.96	14.43 ± 3.95	16.89 ± 6.38	0.029
Median (IQR)	16 (8)	14 (4)	15 (7)	
9HPT	29.15 ± 7.14	26.86 ± 5.88	28.44 ± 6.96	0.107
Median (IQR)	28 (9)	26 (5.9)		
MFIS	16.13 ± 6.38	13.48 ± 4.62	15.31 ± 6	0.03
Median (IQR)	15.31 (1)	15.31 (5)	15.31 (0)	
Cognitive function				
SMDT	27.96 ± 10.89	29.20 ± 8.62	28.37 ± 10.21	0.441
Median (IQR)	28.37 (15)	29 (11)	28.37 (15)	
BVMTR	20.66 ± 8.78	20.55 ± 8.84	20.63 ± 8.75	0.87
Median (IQR)	20.63 (12)	20 (13)	20.63 (12)	
CVLT	40.66 ± 8.79	39.7 ± 8.08	40.36 ± 8.55	0.91
Median (IQR)	39 (11)	39.5 (11)	39 (11)	
Hamilton depression rating scale (HDRS)	11.85 ± 6.77	8.81 ± 5.81	10.9 ± 6.61	0.027
Median (IQR)	10 (8)	7 (10)	10 (8)	
sNfL	83.77 ± 65.06	61.5 ± 34.81	76.85 ± 58.15	0.110
Median (IQR)	73 (64.31)	55.2 (39.34)	68.25 (54.91)	
OCB number	4.28 ± 2.13	3.54 ± 1.56	4.05 ± 2	0.38
Median (IQR)	4 (1)	4 (2)	4 (2)	

The Chi-square test was used for categorical variables. The Mann–Whitney test was used for continuous variables. 9-HPT, 9-Hole Peg Test; 25-FWT, twenty-five foot walking test; BVMT, Brief Visuospatial Memory Test; CIS, clinically isolated syndrome; CVLT, California Verbal Learning Test; DMTs, disease-modifying therapies; EDSS, Expanded Disability Status Scale; HDRS, Hamilton Depression Rating Scale; IQR, interquartile range; MFIS, Modified Fatigue Impact Scale; MRI, magnetic resonance imaging; MS, multiple sclerosis; OCB, Number of oligoclonal band; PPMS, primary progressive multiple sclerosis; PSQI, Pittsburgh Sleep Quality Index; RRMS, relapsing–remitting multiple sclerosis; SDMT, Symbol Digit Modalities Test; sNfL, serum neurofilament light chain; SPMS, secondary progressive multiple sclerosis.

Table 4 presents the Spearman correlations between PSQI scores and various clinical variables in MS patients. The analysis revealed several significant positive correlations with sleep quality. PSQI scores were found to correlate positively with the total number of attacks (r = 0.236, *p* = 0.016), 25-FWT (r = 0.305, *p* = 0.002), the MFIS score (r = 0.244, *p* = 0.013) and HDRS (r = 0.256, *p* = 0.009).

**Table 4:** Spearman correlation between PSQI and different demographic and different rating scales.

Demographic data and different assessment scales	PSQI	
Total number of attacks	r	0.236^*^
	*p* value	0.016
25-FWT	r	0.305^**^
	*p* value	0.002
MFIS	r	0.244^*^
	*p* value	0.013
Hamilton depression rating scale	r	0.256^**^
	*p* value	0.009

25-FWT, twenty-five-foot walking test; MFIS, Modified Fatigue Impact Scale; PSQI, Pittsburgh sleep quality index. * Indicates *p* < 0.05. ** Indicates *p* < 0.01.

Table 5 illustrates the effect of sleep quality on the QoL in patients with MS, as measured by the SF-36 questionnaire. These results imply that MS patients’ poor sleep quality has a wide-ranging detrimental effect on many facets of their quality of life. Perceptions of overall health and emotional well-being, however, showed no difference between groups.

**Table 5:** Impact of sleep disorders on quality of life (SF-36).

SF36 domains	Poor sleep quality (*n* = 71)	Good sleep quality (*n* = 32)	*p*-value
Physical function	60.15 ± 24.74	70.26 ± 22.75	0.05
Median (IQR)	63.29 (35)	70 (35)	
Limited to physical	40.2 ± 35.38	60.2 ± 37.05	0.006
Median (IQR)	46.57 (65)	57.5 (53)	
Limited to emotion	41.64 ± 35.12	60.14 ± 37.87	0.013
Median (IQR)	47.38 (67)	66.55 (66)	
Energy fatigue	44.4 ± 16.36	53.2 ± 16.58	0.035
Median (IQR)	47.13 (15)	48.57 (29)	
Emotion wellbeing	53.58 ± 13.75	57.82 ± 14.71	0.37
Median (IQR)	54.04 (16)	58 (15)	
Social	55.62 ± 14.92	65.2 ± 18.54	0.002
Median (IQR)	58.6 (13)	64 (16)	
Pain	67.31 ± 20.28	78.34 ± 18.98	0.009
Median (IQR)	70.74 (25)	77 (29)	
General health	42.76 ± 17.21	50.2 ± 20.32	0.12
Median (IQR)	45 (20)	45.07 (44)	

IQR, interquartile range; SF-36, Short Form 36 Health Survey Questionnaire.

## 4 Discussion

Our study reveals several crucial insights into sleep disorders in MS patients. The key findings of the present study included a significantly higher frequency of sleep disorders in patients with MS (68.9%) in comparison to controls. Poor sleep quality in MS patients has shown a significant association with the total number of MS relapses, 25-FWT, MFIS scores, and HDRS scores. Importantly, MS patients with poor sleep quality reported lower scores across various aspects of QoL.

### 4.1 Frequency of sleep disorders

In the current study, 68.9% of MS patients experienced poor sleep, compared to 30.6% of healthy controls. This result aligns with prior research (3). The frequency observed in this study is comparable to rates reported in other studies (13, 40–42) but higher than those found in some other studies (3, 43–45). However, it remains slightly lower than a recently reported prevalence of 74.7% (73.7% in women and 76.8% in men) (6). Variations in reported prevalence rates across studies may be attributed to differences in MS populations, sample sizes, definitions of sleep disorders, and methods used to assess sleep quality (6). Additionally, cultural influences, geographical differences, and methodological variations may contribute to these discrepancies (13).

Among MS patients, multiple factors contribute to poor sleep, including MS-related symptoms, specific sleep disorders, and adverse effects of medications (46, 47). The development of sleep disorders has been linked to various immunologic factors in serum. Given that MS is characterized by immune dysfunction, it is reasonable to assume that MS and sleep disorders share underlying mechanisms. However, sleep disorders should be investigated as distinct conditions due to their unique etiopathological pathways (48). Fatigue, one of the most frequent and devastating symptoms of MS, shows a strong bidirectional relationship with sleep disturbances. Research continues to demonstrate the critical role of sleep in managing MS-related fatigue, emphasizing the importance of comprehensive sleep assessment and intervention as part of standard MS care protocols (49).

The geographic location of the present study may have influenced our findings in several ways: Egypt’s hot climate and variable daylight hours could impact sleep patterns, particularly in patients with MS, who often experience heat sensitivity (Uhthoff’s phenomenon). Warmer temperatures may contribute to increased fatigue and nocturnal discomfort, potentially worsening sleep quality. Differences in sleep schedules, daily routines, and napping habits may influence sleep quality. In some Middle Eastern cultures, later bedtimes and shorter nighttime sleep durations are common, which could contribute to the high prevalence of poor sleep in this study. Variability in healthcare access, diagnostic capabilities, and MS management strategies may affect symptom burden, including sleep disturbances (50). Economic and social stressors unique to the Egyptian population may contribute to higher levels of stress-related sleep disturbances. Recent studies suggest genetic and ethnic differences influence MS susceptibility, progression, and symptomatology. Certain genetic factors associated with fatigue, inflammation, or neurotransmitter function may differ between Egyptian MS patients and those studied in Western cohorts, potentially impacting sleep quality.

### 4.2 Poor sleep and demographic variables

Consistent with Sahraian et al. (51), our results did not find any significant differences in medical comorbidities or demographic characteristics between MS patients with and without poor sleep quality. Other research, however, has connected the number of comorbidities to inadequate sleep (44, 52).

In this study, there was no significant difference in the frequency of poor sleep between men and women. This finding aligns with previous studies that reported no gender-related differences in MS patients with poor sleep quality (6, 53, 54). Hence, sleep disorders appear to affect both genders likewise (6). However, some research has suggested that women are more susceptible to experiencing poor sleep than males (3, 41, 43, 55). This variability suggests further research to confirm these findings (3, 6).

### 4.3 Poor sleep and demographic, clinical phenotypes, first clinical presentation, and others in MS patients

In this study, the most common clinical MS phenotypes, first clinical presentation, MRI lesion location, disease duration, severity of EDSS, cognitive function, serum NfL, and OCB showed no differences between good and poor sleepers. Similar results were reported by Sahraian et al. (51), who did not observe any correlation between sleep quality and age, gender, EDSS, or disease duration.

Poor sleep was not significantly related to disease duration in this study, consistent with Zhang et al. (6) and Mosarrezaii et al. (13). Additionally, poor sleep quality did not cluster with common MS symptoms (40). However, Vitkova et al. (43) found longer disease duration was associated with higher rates of poor sleep. Despite no differences in MS phenotype between groups, all SPMS patients in this study experienced poor sleep, echoing findings from Zhang et al. (6), who reported that many SPMS patients had sleep disturbances. However, the present study revealed significantly higher relapse rates among MS patients with poor sleep quality, establishing a notable association between sleep quality metrics and total relapse frequency. This relationship suggests potential bidirectional mechanisms: disease activity may disrupt sleep patterns, or compromised sleep quality might enhance relapse susceptibility (7).

A recent study by Laslett et al. (40) identified recent MS relapse as an independent predictor of poor sleep quality in multivariate analyses. Furthermore, Sahraian et al. (51) proposed sleep disturbance as a potential risk for acute exacerbation in MS, advocating for routine sleep disorder screening and specialist awareness as a therapeutic intervention for sleep disorders might mitigate relapse occurrence. While these findings suggest a meaningful relationship between sleep quality and disease activity, establishing definitive causality would require longitudinal studies.

In this study, while no significant difference was found in disability measured by EDSS, the 25-FWT was significantly impaired in MS cases with poor sleep compared to those with good sleep. Substantial positive associations between PSQI scores and 25-FWT further confirmed that poor walking performance was associated with poor sleep. This means that 25-FWT is more specific than the total EDSS for assessing sleep quality. Several studies have linked poor sleep quality to the severity of disability (6, 56, 57). However, many other studies did not find these relationships (3, 7, 58, 59). This confirms that sleep disturbance is prevalent even among MS patients with low disability levels (60).

### 4.4 Influence of depression and fatigue as confounding factors

Sleep disturbances, depression, and fatigue are closely interconnected in MS, with each factor capable of affecting cognitive function. In our study, poor sleep quality was significantly associated with higher depression (HDRS) and fatigue (MFIS) scores, suggesting that these symptoms are prominent among poor sleepers. Some prior studies have suggested that self-reported sleep disturbances in MS may reflect underlying mood disorders (3, 41, 61) rather than true sleep-related cognitive dysfunction. This could explain why sleep disturbances in our cohort were not directly linked to cognitive impairment, as depressive symptoms and fatigue (3, 41, 52, 61) may have been more influential factors.

Kotterba et al. (61) further corroborated these associations, demonstrating that poor sleepers exhibited diminished performance on MFIS and SF-36 scales and that poor sleep quality positively correlates with fatigue and reduced functional health status. The factors contributing to poor sleep quality in MS patients are similar to those in the general population. These factors include increased fatigue, severe pain or urinary dysfunction, and elevated depression and or anxiety levels (41, 43, 54, 61).

Sleep disturbance is a critical mediator of symptoms associated with multiple sclerosis, with extensive relationships between sleep, pain, and fatigue (10). Fatigue, which affects up to 90% of patients with MS, represents a leading contributor to unemployment, early retirement, and disability (62, 63). Recent meta-analytic evidence from Bhattarai et al. (64) suggests that insomnia and subjective sleep quality perceptions demonstrate stronger associations with fatigue than objective sleep duration measurements, potentially indicating more effective intervention targets in MS management.

Depression affects up to 50% of individuals with MS, which is 2–3 times more prevalent than in the general population (65). Depression and sleep have a well-documented bidirectional relationship, with psychological disorders directly correlated with impaired sleep patterns and poor sleep hygiene (66). Zhang et al. (6) emphasize that poor sleep quality may exacerbate fatigue and depressive symptoms, while these conditions may reciprocally impact sleep quality.

### 4.5 Poor sleep and cognitive function and potential reasons for discrepancies with previous studies

Sleep disturbances in MS have been linked to impairments in various cognitive domains, such as attention, different types of memory, and processing speed (67). Additionally, patients with sleep disorders have more subjective cognitive problems than those with normal sleep (68).

In the present study, both groups maintained cognitive performance above threshold values across three assessment instruments, with no significant differences between poor and good sleepers. These findings align with previous meta-analysis (69), as they reported no significant associations between sleep quality and SDMT performance. While these results suggest poor sleep may not reliably predict cognitive deficits, contrasting evidence from multiple other studies evaluated in Golabi et al.’s (69), Hughes et al. (70) reveal potential associations between sleep quality and cognitive performances across various assessment scales.

Several factors may explain these discrepancies: firstly, differences in cognitive assessment tools; as the present study utilized the SDMT, BVMT-R, and CVLT-II, which primarily assess processing speed, visuospatial memory, and verbal learning. However, previous studies reporting stronger associations between sleep and cognitive impairment have often used broader neuropsychological batteries that include assessments of executive function, working memory, and sustained attention. Sleep disturbances in MS may have a greater impact on these specific cognitive domains, which were not the primary focus of our study. Secondly, cognitive impairment definitions vary across studies. Some research categorizes impairment based on a single affected domain, while others require deficits in multiple domains. Our study used established thresholds to define cognitive dysfunction, but subtle cognitive changes that do not meet these cutoffs may still be present and influenced by sleep disturbances. Another explanation related to differences in patient populations as cognitive function in MS is influenced by disease duration, lesion burden, and overall disease severity. Our cohort consisted of relatively young patients with relatively short disease duration, which may explain the preserved cognitive function observed. In contrast, studies reporting stronger links between sleep and cognition often include patients with longer disease duration, higher disability scores, or progressive MS phenotypes, where cognitive decline is more pronounced. Furthermore, all patients in our study had cognitive performance above the established impairment thresholds, suggesting that our cohort may not yet exhibit significant cognitive decline. This could explain why sleep disturbances did not appear to have a measurable impact on cognition in our sample.

Our study relied on the PSQI, a validated but subjective sleep assessment tool. Previous studies that found stronger correlations between sleep and cognition often included objective measures such as polysomnography (PSG) or actigraphy, which assess sleep architecture, sleep fragmentation, and specific sleep stages (e.g., slow-wave sleep, REM sleep). These parameters are known to be crucial for memory consolidation and cognitive performance. It is possible that while our patients reported poor sleep quality, their actual sleep architecture may not have been sufficiently disrupted to impact cognition significantly.

### 4.6 Impact of sleep disorders on QoL among patients with MS

In this study, the analysis of SF-36 scores demonstrated that MS patients with poor sleep quality experienced poor QoL across multiple domains, highlighting the profound impact of sleep problems on their overall well-being. In the current study, the SF-36 was specifically used as it does not refer to sleep disorders, thus corroborating with statistical evidence the burden of sleeping disorders on dimensions such as social function, body pain, physical health limitation, emotional limitation, and energy fatigue. Several studies in the literature support these findings. Ma et al. (3) evaluated QoL using the MS Impact Scale (MSIS-29) and found that MS patients with elevated physiological and psychological scores were more susceptible to developing poor sleep patterns. Moreover, Laslett et al. (40), using the Assessment of Quality-of-Life 8-D, established that poor sleep quality was prevalent among MS patients and showed a strong independent association with reduced QoL, distinct from other MS symptoms. Their findings suggest that sleep quality improvement could be a crucial intervention target for enhancing overall QoL in MS patients. Building on this evidence, Kołtuniuk et al. (9) identified specific sleep disturbances, particularly insomnia and daytime sleepiness, as significant determinants of QoL in MS patients. They emphasized that maintaining regular sleep patterns, adequate sleep duration, and minimizing sleep disturbances are essential for optimal QoL outcomes.

### 4.7 Clinical implications and future directions

The high frequency of sleep disorders in our MS cohort and their association with various symptoms and QoL measures emphasize the necessity for routine sleep assessment in MS care. Given the strong link between sleep disturbances and key MS symptoms such as fatigue, depression, and cognitive impairment, integrating sleep evaluation into regular neurological assessments is crucial. Clinicians should implement validated screening tools, such as the PSQI or the Epworth Sleepiness Scale (ESS), during routine visits to promptly identify sleep disturbances.

A multidisciplinary approach is essential for managing sleep disorders in MS. Cognitive-behavioral therapy for insomnia (CBT-I) should be considered a first-line intervention, alongside sleep hygiene education, regular physical activity, and mindfulness-based techniques. Pharmacological options, such as melatonin or non-sedative sleep aids, may be used cautiously when necessary. Addressing underlying contributors like pain, spasticity, and medication side effects should also be prioritized.

### 4.8 Strength of the study

Unlike some previous studies that relied on retrospective data or patient-reported surveys without supervision, our study employed a cross-sectional design with structured, supervised data collection at a dedicated MS unit. Many past studies used convenience samples or registry data, whereas our study recruited patients consecutively from a specialized MS unit, reducing selection bias.

The present study included patients across different MS phenotypes (CIS, RRMS, SPMS, and PPMS), whereas many previous studies focused primarily on RRMS. The inclusion criteria ensured that participants were in a stable disease phase (no relapses for 3 months), which minimizes confounding effects of acute disease activity on sleep. Patients were diagnosed using the 2017 McDonald Criteria, ensuring a standardized and up-to-date approach to MS classification.

Many studies on sleep in MS have used either a single assessment tool or focused primarily on insomnia or daytime sleepiness. Our study, however, used the Pittsburgh Sleep Quality Index (PSQI) to evaluate multiple sleep domains and correlated these findings with: Neurological disability (EDSS, 25-FWT, 9-HPT), fatigue (MFIS), Depression (HDRS), Cognitive function (SDMT, BVMT-R, CVLT-II) and Quality of life (SF-36). This multi-dimensional approach provides a more detailed and clinically relevant picture of sleep disturbances in MS.

Analysis of Disease-Modifying Therapies (DMTs): the present study classified patients based on DMT use and efficacy levels, allowing for insights into how different MS treatments may (or may not) influence sleep quality. Some prior studies did not distinguish between treatment-naïve and treated patients, making it difficult to assess potential medication effects on sleep. Geographic and Cultural Differences: The present study is among the first to comprehensively assess sleep quality in an Egyptian MS population. The demographic, genetic, and environmental factors unique to this population may contribute to different sleep disturbance patterns compared to studies conducted in Western or Asian populations.

### 4.9 Study limitations

This study has several limitations. The cross-sectional design presents inherent limitations, preventing a definitive temporal understanding of sleep problems in MS patients. Furthermore, the research lacks comprehensive information about potential comorbidities that might influence sleep patterns and other clinical factors. The reliance on subjective sleep measures (PSQI) without objective sleep data (e.g., polysomnography) may not capture the full spectrum of sleep disturbances (49). Furthermore, this study did not extensively analyze the influence of specific MS medications on sleep quality.

## 5 Conclusion

Our findings demonstrate that sleep problems are remarkably common among individuals with MS. They also demonstrate significant associations between sleep problems and various MS symptoms, including the number of relapses, walking, fatigue, and depression. The effect of poor sleep on QoL in MS patients is substantial and multifaceted. These results highlight the importance of including sleep assessment and management in standard MS care to enhance overall patient outcomes and QoL.

### 5.1 Recommendation

Future research should prioritize longitudinal studies to determine causal relationships between sleep disorders and MS symptoms. Additionally, interventional studies examining the efficacy of non-pharmacological treatments, such as tailored sleep rehabilitation programs or personalized sleep therapy, could provide valuable insights into optimizing disease management and improving QoL in MS patients.

## Data Availability

The original contributions presented in the study are included in the article/supplementary material, further inquiries can be directed to the corresponding authors.
